# Commentary: Physical time within human time

**DOI:** 10.3389/fpsyg.2023.1085761

**Published:** 2023-08-03

**Authors:** Barry Dainton

**Affiliations:** Department of Philosophy, University of Liverpool, Liverpool, United Kingdom

**Keywords:** time-consciousness, specious present, externalism, presentism, snapshot, stream of consciousness

## Time and illusion

For Callender the two times problem is more serious than the problem posed by Eddington's two tables. The two tables do appear very different: the solid colored object that we can see and doesn't seem to be mostly empty space. However, in this case we have the beginnings of a plausible story about how the manifest table emerges from its basic ingredients, but we don't have this in the case of time. Our everyday experience suggests that the present is special and very different from the past and future. If we take physics as our guide none of the ingredients of manifest time are to be found in real time: “our best science of time suggests that manifest time is more or less rubbish” (Callender, 2017, p. 2).

Callender finds the size of the gap between manifest time and physics-based time disturbing, and sets himself the goal of establishing that it's at least *intelligible* that manifest time could emerge if the physics-inspired model of time is correct. His project is one of de-mystification. In their “Physical Time Within Human Time” Gruber, Block, and Montemayor (GBM) find a lot to like in Callender's project, but adopt a somewhat different goal (see Gruber et al., 2022). Their dualistic approach seeks to isolate those aspects of experience which correspond to real features of reality and those illusory aspects which don't. By showing that some aspects of manifest time are *not* illusory they hope to console.

About one thing GBM are under no illusions: the complexity and difficulty of their project. Some of these difficulties derive from ongoing disagreements about the nature of temporal experience, but others derive from physics, where there the nature of time continues to be hotly debated. In a recent paper (Gruber et al., 2020) the same authors heroically considered a total of 10 different spacetime cosmologies, many of them providing very different conceptions of time. Unfortunately Callender's “best science of time” is still a long way from having a settled story on what time really is.

The project of Buonomano and Rovelli in “Bridging the Neuroscience and Physics of Time” is different again (Buonomano and Rovelli, 2021). They suggest that a necessary first step is to acknowledge that temporality is multifaceted and has a number of different aspects. After outlining some of the more important they propose a division of labor, with some problems going to physics for solutions and the remainder to neuroscience. Physics has the job of discovering “the general temporal structure of the world” along with additional temporal features that become relevant at biological scales. Neuroscience has the task of explaining all the other features, such as the apparent difference between past and future, the (seemingly) special role of the present, memory and why time seems to flow.

## Which science?

Buonomano and Rovelli agree on a good deal but they also disagree on one big issue: the nature of time, with Rovelli leaning strongly to the eternalist view that past, present, and future are all equally real, and Buonomano finding local presentism more plausible—on this view reality is confined to the here and now, and the past and future don't exist. Since I see the appeal of each of these positions I see nothing to criticize here. However, presentism and eternalism are surely contrasting positions on the *general temporal structure of the world*. Given this, I wondered whether they fully share the view that discovering the temporal structure of reality is solely the task of physics. The appeal of presentism is rooted in those features of our everyday experience which can make it seem *just obvious* that we live out our lives in a brief window of presence that is a steadily advancing, and that present things are real in a way that other things are not. Presentists are (typically) prepared to give primacy to features of the manifest world—even if this means rejecting what physics has to say. I suspect Buonomano is similarly motivated.

Buonomano has further reasons for finding eternalism problematic. These reasons are in fact scientific, but the relevant sciences are evolutionary biology and neuroscience. From an evolutionary perspective it would be odd if our feeling that time is dynamic lacks any survival value. But this would be the case if the eternalists are right and our experience of flow is illusory. Buonomano also suggests that much of the appeal of eternalism derives from peculiarities of the human brain that science has revealed. Like Bergson before him, Buonomano holds that our innate preference for spatial modes of thinking may well be misleading us about the nature of reality. More specifically, this spatializing tendency makes the four-dimensional conception of time more appealing that it would otherwise be.^1^

The Buonomano-Rovelli exchange serves as a useful reminder that while physics has an important role to play, when it comes to understanding time physics is not the only science that matters.

## Streams and structures

These debates aren't confined to the sciences: philosophers have also long been engaged in debates concerning the nature of time and temporal experience. For better or worse, they are as far from reaching agreement on these topics as the physicists. Much of my own work in this area has been focused on temporal experience and the structure of our streams of consciousness. GBM make some claims about these topics which struck me as questionable.

Our sense that time is something that flows has several components, but a centrally important one is the experiential (or specious) present, that brief experienced interval during which we directly apprehend change and persistence. It's here that consciousness is at its most vividly dynamic. On the view I find most plausible the experiential present is a single experience whose successive parts are experientially unified, and which extends through ordinary physical time in much the way it seems to. How much time? It's not easy to be precise, but not much: a single second, probably less. Smolin and Varde concur: “The moments of awareness seem to define a thick present. There is also a duration of each experienced moment in time of about 0.5 of a second” (Smolin and Verde, 2021, §5).

Drawing on Pöppel's work GBM suggest that the duration of the experienced present is significantly longer than this: 3 s or so. If we take the experienced present to be a single unified episode of experience this strikes me as implausible. The main reason for this is simple: my own direct experiences of change simply don't seem to last anything like that long. If I clap my hands three times, at roughly one clap per second, by the time I hear the third clap I am no longer experiencing the first.^2^

There is a further important element of our ordinary temporal experience: *continuity*. If I listen to a succession of brief notes each note has its own short duration and each note is experienced as giving way to the next. In the case of the sequence (C-D-E), I hear (C-followed-by-D) and (D-followed-by-E). Here too there is a simple and plausible way of making sense of this: successive experiential presents *partially overlap*. Accordingly, in our current example we have two experiential presents (C-D) and (D-E), where the D-note in the first is numerically identical with the D-note in the second. This form of continuity is not confined to the auditory sphere, it is—I argue—found throughout our streams of consciousness. I have defended this “extensional” view of temporal experience on a number of occasions—see Dainton (2000, 2008, 2016, 2017), and in differing guises it has found favor with others, see Hoerl (2009), Rashbrook (2013), Phillips (2014), Piper (2019), and Dorato and Wittmann (2020).

## Snapshots or streams?

In their closing section on possible tests for their model GBM venture that “the dualistic view predicts an existence of a discrete (snapshot) perception in the absence of the specious present.” Defenders of the snapshot (or cinematic) view hold that our streams of consciousness consist of successions of momentary experiential phases that possess static motion-free contents, and that are also entirely distinct from one another. They deny that we directly experience change, and hence deny that specious or experiential presents—at least in the extensional guise of just outlined. I think GBM should pause and reconsider before embracing this view.

While it has some defenders, in the recent philosophical debates the snapshot theory is also widely seen as problematic. This is largely because (a) since our consciousness seems continuous and we do seem to experience change it is phenomenologically suspect, and (b) doubts about the empirical evidence cited in its favor. In this connection GBM mention the wagon-wheel illusion; but—as they acknowledge—there are competing interpretations which point in a different direction. They also point to Arstila's (2018) defense of a dynamic snapshot model. Arstila has suggested that snapshot theorists can appeal to the waterfall illusion order to explain how durationless experiences can seem dynamic without really being so. However, this move has itself come under sustained critical fire recently: Shardlow (2019) and McKenna (2020) find it flawed on several grounds.

If the snapshot view is problematic it is regrettable that something like it has been widely assumed in much scientific work on consciousness.^3^ Northoff and Lamme (2020) review eight of the main neuroscientific theories of consciousness: global neuronal workspace theory (GNWT), predictive coding theory (PCT), embodied theory (EB), temporospatial theory of consciousness (TTC), integrated information theory (IIT), recurrent processing theory (RPT), synchrony theory (ST), and higher-order thought theory (HOT). Drawing on this Kent and Wittmann (2021) argue that nearly all of these theories have thus far assumed that our temporal experience is confined to isolated brief 100–300 ms phases duration. As a result these theories have all confined themselves (in effect) to experienced momentary *simultaneity*, they have nothing to say about experienced *succession*, and so all are fatally flawed. In a similar vein Singhal et al. (2022) criticize IIT for failing to recognize that unity of consciousness extends through time and they recommend an addition to IITs existing axioms: “experience always occurs to us as a temporal whole, i.e., experience always has an extension, is continuous and has an inherent direction that is asymmetric” (Singhal et al., 2022, p. 14). I couldn't have put it better myself—though we should also remember that we need an account of how these individual experiential presents combine to form streams of consciousness.

A final quick thought. On one issue Buonomano and Rovelli are in full agreement with one another: if a time traveler from the future were to arrive we could be certain that the eternalist conception of time is correct. You can't arrive from a location that doesn't exist, and presentists hold that the past and future don't exist. For better or worse, as things currently stand time travelers are confined to the realm of fiction. But there might be empirical evidence of a different sort that's relevant to the debate between presentists and eternalists.

Just as it is likely that there will always be some people who give primacy to their everyday experience of temporality when deciding on the view of time that is most plausible, there are also people who adopt the same policy when it comes to the nature of perception. Since the days of Galileo the scientifically respectable view has been that when you look at a red apple sitting on the table in front of you the resulting perceptual experience is some kind of brain-generated inner mental representation, and the redness resides not on the apple's surface but in your consciousness. But it certainly doesn't seem that way: it seems (very much) as though I am directly aware of *the apple itself*. For proponents of the “direct (or naive) realist” account of perception Galileo was wrong, and seeing works in the way it seems: colors really are outside in the world, rather than in our heads. Among contemporary philosophers of perception direct realism is certainly not the dominant view—see Crane and French (2021) for an overview—but it still has its defenders.

One objection to direct realism runs along these lines. We only see distant objects when light emitted by them reaches our eyes. In the case of distant stars or galaxies, the relevant light may have been traveling thousands or millions of years. Isn't it absurd to think we could be directly aware of an event in the distant past or an object which no longer exists? Direct realists do not take this to be an insuperable problem. As A. J. Ayer noted, this objection presupposes that we can only see what is present, but perhaps this assumption is wrong: “Why should it not be admitted that our eyes can range into the past, if all that is meant by this is that the time at which we see things may be later than the time when they are in the states in which we see them? And having admitted this, then should we also not admit that it is possible to see things which no longer exist?” (Ayer, 1982, p. 94–95). For a more recent defense of this position with regard to the perception of the past see Manzotti (2017 chapter 7, 2019).

If the direct realists are right and we are directly aware of past events then it can scarcely be denied that these past events are real. If the past is real then presentism is false. Moreover, presentism has been falsified by ordinary perceptual experience rather than the arrival of a time traveler. Of course, you may not find the direct realist view of perception an appealing one. But it's still of some interest to find out that two important ingredients of the manifest world—presentism and direct realism—are not compatible with one another.

## Author contributions

The author confirms being the sole contributor of this work and has approved it for publication.

## Footnotes

^1^This topic is explored in more detail in Buanomano (2017, chapter 10).

^2^There's evidence of a different sort: in a recent comprehensive survey of relevant empirical work (White, 2017) finds little evidence to support the three seconds proposal, stating in his conclusion: “There is no three-second subjective present or experienced moment. There is, instead, an envelope of integration in which there are multiple qualitatively different representations of what is going on, each occupying different and variable time scales of integration…”.

^3^Widely but not universally—see Northoff (2016) and Piper (2019) for neuroscientific approaches that reject the snapshot view and focus on temporally extended neural processes that are compatible with extensionalism.
